# Field‐applicable low‐intensity exercise induces bronchodilation in horses with severe asthma

**DOI:** 10.1111/evj.70111

**Published:** 2025-11-04

**Authors:** Sophie Mainguy‐Seers, Sarah‐Maude Grondin, Jean‐Pierre Lavoie

**Affiliations:** ^1^ Department of Clinical Sciences Faculty of Veterinary Medicine, Université de Montréal Saint‐Hyacinthe Quebec Canada

**Keywords:** heaves, horse, lung function, physical training, salbutamol, β2‐adrenergic agonist

## Abstract

**Background:**

Airway dysfunction in severe equine asthma (SEA) often results in early retirement or euthanasia of affected horses. Exercise‐induced bronchodilation occurs in horses with SEA after intense treadmill exercise, but the effects of a lighter, field‐applicable, training regimen remain largely unexplored.

**Objectives:**

To evaluate the impact of submaximal aerobic exercise on airway obstruction during exacerbation of SEA.

**Study Design:**

The preliminary phase explored the effects of a 25‐min standardised exercise on the lung function of eight SEA horses. As notable bronchodilation occurred, the results were confirmed in a randomised controlled crossover protocol comparing the effect of the same standardised exercise on lung function with that of an equal duration turnout.

**Methods:**

Lung function was assessed by standard lung mechanics before and 15 min after the interventions (turnout and walk/trot lungeing exercise). To compare exercise‐induced bronchodilation with that of a potent bronchodilator in the main study, salbutamol was administered after the turnout intervention only, and lung function was measured again 5 min later. Data were analysed with *t* tests, Wilcoxon tests, and mixed‐effects models.

**Results:**

In the preliminary phase, exercise led to a mean 50% reduction in pulmonary resistance. This was confirmed in the main study, where pulmonary resistance decreased from 2.6 (2.1–3.3) to 1.3 cm (0.8–2.2) H_2_O/L/s after exercise (*p* = 0.006), whereas it remained unchanged after the turnout intervention. Following salbutamol administration after the turnout, lung function was comparable to that of the lunged horses, suggesting that the extent of exercise‐induced bronchodilation was similar to that achieved with a β2‐adrenergic agonist.

**Main Limitations:**

The small number of aged horses, the lack of blinding, and the focus on the short‐term effects of exercise restrict the generalisability of the results.

**Conclusions:**

The rapid and potent bronchodilation achieved through brief aerobic exercise supports the investigation of a training program's impact on the management of SEA.

## 1 INTRODUCTION

Severe equine asthma (SEA) is an incurable disease that impairs exercise tolerance and compromises welfare, representing a major concern for horse owners.^1^ Although common pharmaceutical interventions (corticosteroids and bronchodilators) are highly effective in improving airway obstruction, their benefits are short‐lived. Also, they can be associated with adverse effects,^2^ and come at a cost for horse owners. Ancillary treatments to improve the respiratory health and well‐being of horses with severe asthma are therefore needed.

In human asthmatics, physical training improves symptoms, quality of life, lung function, and global health,^3^, ^4^ even though up to 90% of asthmatic patients experience acute bronchoconstriction after exercise.^5^ While this phenomenon can also occur in healthy individuals and elite athletes, the physiological response to physical activity is bronchodilation.^6^, ^7^ Similarly, exercise causes bronchodilation in healthy horses,^8^, ^9^, ^10^ although this is not universally accepted.^11^, ^12^ While the effects of exercise in equine asthma are largely unexplored, previous reports have shown the occurrence of bronchodilation in horses with SEA that are run up to fatigue on a treadmill.^13^, ^14^ Because affected horses appear to retain their physiological response to exercise, unlike human asthmatics, species‐specific studies are required to determine whether physical activity could contribute to disease management.

As the possibility of exercising severely asthmatic horses could improve their quality of life, overall health, and influence owners' decisions to keep these animals,^1^ the aim of this study was to assess the impact of a single field‐feasible lungeing exercise on airway obstruction in horses with SEA, with the hypothesis that a low‐intensity aerobic activity would induce bronchodilation.

## 2 MATERIALS AND METHODS

### 2.1 Animals

As previous studies have shown a relief of airway obstruction following strenuous exercise in fewer than six horses with severe asthma,^13^, ^14^ 10 horses from a research herd were studied in a preliminary phase to account for a possible milder bronchodilation with light exercise. The horses were diagnosed with SEA based on the historical development of airway obstruction (pulmonary resistance [R_L_] >1 cm H_2_O/L/s) and bronchial inflammation (>25% neutrophils in bronchoalveolar lavage fluid) when exposed to hay. The horses had been part of the research herd for 2–9 years, during which their physical activity was limited to daily turnout in a pasture or paddock. All horses were lunged and trotted 1 month before the start of the study to evaluate the presence of lameness. Horses with a lameness grade >2/5 (American Association of Equine Practitioners lameness scale) were to receive phenylbutazone (2.2 mg/kg orally) the night before and the morning prior to exercise, while horses with a lameness grade of 4 or higher were excluded. All horses had access to daily turnout in a dirt paddock for the duration of the study.

### 2.2 Exercise protocol

The lungeing exercise was performed outside on a dirt or grass paddock and consisted of 5 min of walking (2.5 min in each direction), 7.5 min of trot in one direction, 2 min of walking, 7.5 min of trot in the other direction, and 3 min of walk. Speed was not measured, and horses were allowed to choose their preferred pace.

### 2.3 Preliminary phase

This preliminary phase took place at the end of March 2024 and aimed to determine the feasibility and safety, as well as to confirm the onset of bronchodilation in SEA horses after exercise. Horses had been exposed to hay starting 9 weeks prior to this study to induce exacerbation for another project. They were exposed for 4 weeks, and then, 5 weeks before the start of the current study, they were fed oiled‐mixed hay (Nutrifoin) to progressively improve their lung function.^15^ Therefore, they were experiencing varying degrees of asthma exacerbation at the time of the study. Lung function was assessed just before and 15 min after exercise. Salbutamol (Apotex) was administered immediately afterward (1000 μg, by inhalation using an Aerohippus device [Trudell Animal Health]), and lung function was measured 5 min later (20 min after exercise) to assess the presence of residual bronchospasm. Blood lactates were measured before and immediately after exercise (Lactate Plus Meter, Nova Biomedical) from blood collected via jugular venipuncture. Heart rate was monitored by auscultation before, after the trot, immediately after exercise, and every 5 min until 15 min post‐exercise. Respiratory rate was visually assessed before exercise, immediately afterward, and again 15 min later.

### 2.4 Main study

As the preliminary phase suggested a clinically relevant bronchodilation with exercise in horses in exacerbation, the study was repeated with a control intervention to confirm that this finding was related to the exercise and not to the experimental procedures. This phase occurred between late April and early May 2024, 1 month after the preliminary phase. Horses were fed varying amounts of hay to maintain a moderate degree of clinical exacerbation according to their individual sensitivity (the remainder of their diet being oiled‐mixed hay). Using a crossover design, each horse was assigned by random digital allocation to perform first the exercise intervention (same as in the preliminary phase), or a 25‐min turnout period in the same paddock. The interventions were reversed after a 1‐week washout period. Lung function was measured before and 15 min after the interventions. Immediately following the 15‐min measurement, salbutamol was administered only to horses that underwent the turnout intervention, and lung function was reassessed 5 min later in both groups to compare the magnitude of exercise‐induced bronchodilation with that achieved using a β2‐adrenergic agonist. Heart and respiratory rates were monitored before, immediately after, and 15 min after the interventions. The observers were not blinded.

### 2.5 Lung function measurement

Lung function was measured in standing and unsedated horses by standard lung mechanics as previously described.^16^ The oesophageal pressure, used as an index of the pleural pressure (P_L_), was obtained with a balloon sealed over the end of a polyethylene catheter positioned in the distal third of the oesophagus and connected to a differential pressure transducer. Flow rates were obtained with a heated pneumotachograph and a differential pressure transducer fitted to a mask placed over the horse's nose. The system (Flexiware 7.6, SCIREQ) electronically integrated the flow signal to provide tidal volume. The R_L_ and elastance (E_L_) were obtained with the multiple regression equation for the single compartment model of the lung $\left(P_{L}=E_{L}V+R_{L}\dot{V}+K\right)$, where V is the volume, $\dot{V}$ the airflow, and K the transpulmonary end‐expiratory pressure.

### 2.6 Statistical analysis

The normality of the data was evaluated with Shapiro–Wilk tests. In the preliminary phase, the effect of exercise on lung function and blood lactate was evaluated with paired *t* tests, or Wilcoxon signed‐rank tests. For statistical analysis of lactate concentrations, values below the lower limit of the test range (0.3 mmol/L) were replaced with a value equal to the limit of detection divided by √2 (i.e., 0.2 mmol/L). Since heart and respiratory rates were assessed serially over time in the preliminary phase and were not normally distributed, they were analysed using Friedman tests for repeated measures followed by Dunn's multiple comparison tests. Lung function and clinical data from the main study were analysed with a mixed‐effects model with time, interventions and the interaction between those factors as the fixed effects. When a significant factor was identified in the mixed‐effects model, multiple comparisons were evaluated with Bonferroni corrections. The effect of the bronchodilator administration on lung function parameters was evaluated with paired *t* tests or Wilcoxon signed‐rank tests in both phases. Data are presented with the mean and standard deviation or with the median and interquartile range if not normally distributed. A *p* value <0.05 was considered statistically significant. GraphPad Prism 10 (GraphPad Prism 10 (version 10.5.0), GraphPad Software, Inc.) was used for statistical calculations.

## 3 RESULTS

### 3.1 Animals

A total of 10 horses were studied, including 3 Quarter Horses, 3 Paint Horses, 1 Canadian, 1 Standardbred, 1 Thoroughbred, and 1 Arabian cross. There were six mares and four geldings, which weighed 532 ± 55 kg and were aged 19.9 ± 2.5 years. Two horses were lame when evaluated before study initiation (one mare with a grade 3/5 chronic lameness of the right hindlimb and one mare with stiffness of both front limbs at the trot, which had no impact on their daily activity).

### 3.2 Preliminary phase

#### 3.2.1 Clinical parameters

Eight horses were in clinical exacerbation (R_L_ >1 cm H_2_O/L/s) and included in this phase. One of the lame mares was included and received 2.2 mg/kg phenylbutazone orally the night before and the morning prior to exercise, which improved the lameness. All horses tolerated the exercise well, although two other horses became lame by the end of the 25‐min exercise. Heart rate increased over time (*p* < 0.0001) and returned to baseline within 10 min after exercise. The respiratory rate increased after exercise (*p* = 0.007; Table 1). Lactate levels remained unchanged (from 0.33 ± 0.13 to 0.36 ± 0.15 mmol/L, *p* = 0.5). Although this was not objectively quantified, all horses willingly performed the entirety of the exercise, requiring minimal encouragement.

**TABLE 1:** Clinical data before, during, and after a 25‐min exercise in horses affected by severe asthma in the preliminary phase.

	Heart rate (bpm)		Respiratory rate (rpm)	
	Median (IQR)	*p*	Median (IQR)	*p*
Before exercise	36 (33–42)		18 (16–31)	
After trot	80 (76–99)	<0.0001		
Immediately after exercise	62 (53–67)	0.004	32 (22–39)	0.09
5‐min post‐exercise	56 (48–59)	0.01		
10‐min post‐exercise	48 (45–48)	0.1		
15‐min post‐exercise	42 (40–44)	>0.99	20 (14–27)	0.8

*Note*: *p* value relates to the difference compared to baseline (before exercise).

Abbreviation: IQR, interquartile range.

#### 3.2.2 Lung function

The 25‐min exercise regimen reduced P_L_ (25.7 [16.6–37.1] to 10.6 [8.8–22.1] cm H_2_O, *p* = 0.008), R_L_ (2.2 [1.6–3.6] to 1.0 [0.6–1.8] cm H_2_O/L/s, *p* = 0.008) and E_L_ (1.9 [0.8–3.2] to 0.8 [0.6–1.6] cm H_2_O/L, *p* = 0.02). Notably, *n* = 4/8 horses had normalised lung function (R_L_ <1 cm H_2_O/L/s) after exercise.

**FIGURE 1:**
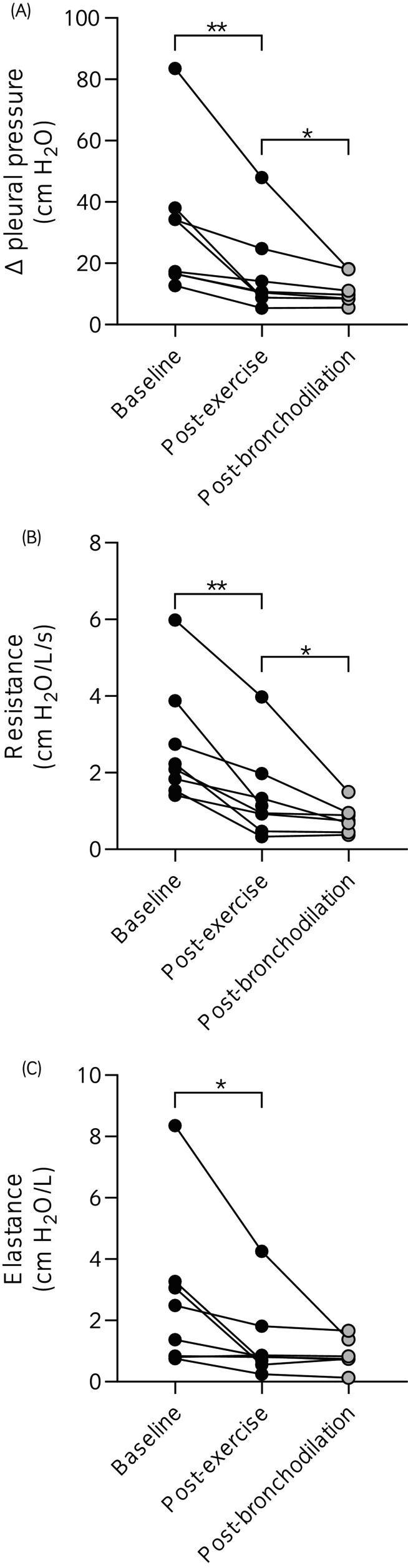
Effect of exercise followed by bronchodilation on lung function in the preliminary phase. Dot plot illustrating the measurement of difference in pleural pressure (A), pulmonary resistance (B), and elastance (C) before and 15 min after exercise, and then following inhalation of a β2‐adrenergic agonist (bronchodilation) in eight severe asthmatic horses. *p < 0.05, **p < 0.01.

One gelding did not complete the bronchodilation test with salbutamol due to a lack of cooperation. The administration of the β2‐adrenergic agonist at 15 min post‐exercise further improved P_L_ (10.6 [8.8–22.1] to 9.7 [8.5–18.1] cm H_2_O, *p* = 0.03) and R_L_ (1.0 [0.6–1.8] to 0.8 [0.4–1.0] cm H_2_O/L/s post‐bronchodilatation, *p* < 0.05), while elastance remained unchanged (0.8 [0.6–1.6] to 0.8 [0.7–1.4] cm H_2_O/L post‐bronchodilation, *p* = 0.2; Figure 1). Excluding the data from the mare that received phenylbutazone did not affect the significance of exercise‐induced reductions in P_L_, R_L_, and E_L_; however, the further improvement with salbutamol lost significance, likely due to reduced statistical power.

### 3.3 Main study

Eight horses were included in this second phase (six were the same as in the preliminary phase), and two were excluded (one mare had a foot abscess and one mare was not in clinical exacerbation). As two additional horses were lame temporarily at the end of the exercise period in the preliminary phase (in addition to the two that were lame prior to the study), all horses received phenylbutazone (2.2 mg/kg orally) the night before and the morning before both exercise and turnout for ethical reasons and to reduce bias. One gelding was excluded from the turnout intervention due to a lack of cooperation for lung function measurements.

#### 3.3.1 Clinical parameters

All horses subjectively tolerated the exercise well, although cough was observed in *n* = 3/8 horses. Heart rate increased over time in both groups (*p* < 0.0001). The residuals for the respiratory rate data in the mixed‐effects model were not normally distributed, so the data were log (10)‐transformed, which normalised the distribution. There were time (*p* = 0.02) and intervention (*p* = 0.01) main effects, and an interaction between time and intervention (*p* = 0.01) for the respiratory rate, as it only increased when horses were exercised (Table 2).

**TABLE 2:** Clinical data before and after a 25‐min exercise or turnout in horses affected by severe asthma.

	Heart rate (bpm)				Respiratory rate (rpm)			
	Turnout		Exercise		Turnout		Exercise	
	Median (IQR)	*p*	Median (IQR)	*p*	Median (IQR)	*p*	Median (IQR)	*p*
Baseline	36 (36–40)		40 (36–40)		20 (16–20)		20 (16–20)	
Immediately after intervention	52 (44–68)	0.0003	62 (53–67)	<0.0001	20 (20–24)	>0.99	32 (25–35)	0.0005*
15‐min post‐intervention	44 (44–48)	0.3	46 (41–51)	0.1	20 (16–20)	>0.99	22 (20–28)	0.4

*Note*: *p* value relates to the difference compared to baseline. *Significantly different between turnout and exercise at the same time point (*p* = 0.0007).

Abbreviation: IQR, interquartile range.

#### 3.3.2 Lung function

**FIGURE 2:**
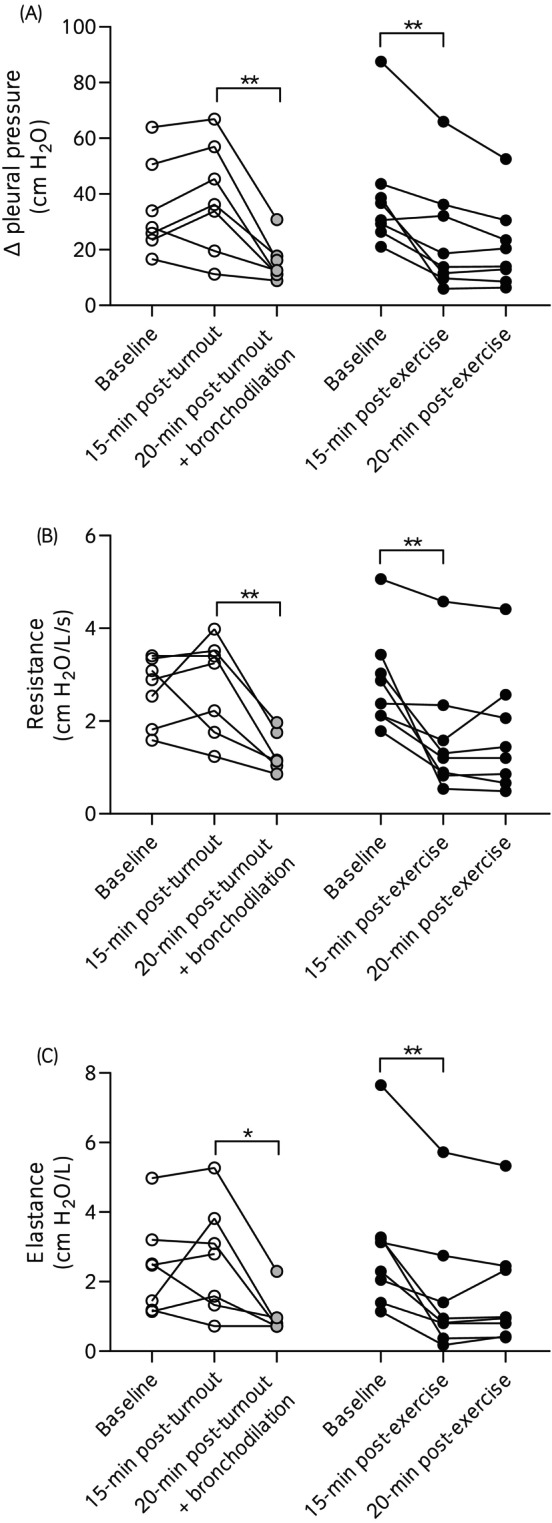
Lung function in the randomised controlled trial. Dot plot illustrating the difference in pleural pressure (A), pulmonary resistance (B), and elastance (C) before and 15 min after a 25‐min exercise, compared to a turnout of the same duration in eight severe asthmatic horses, and after salbutamol administration in turnout horses. *p < 0.05, **p < 0.01.

There was an interaction between time and intervention for P_L_ (*p* = 0.01), R_L_ (*p* = 0.04), and E_L_ (*p* = 0.03), as those parameters only improved with exercise (Figure 2). After exercise, there was a decrease in P_L_ (33.7 [27.1–42.4] to 16.2 [10.2–35.2] cm H_2_O, *p* = 0.002), in R_L_ (2.6 [2.1–3.3] to 1.3 [0.8–2.2] cm H_2_O/L/s, *p* = 0.006), and in E_L_ (2.7 [1.6–3.3] to 0.9 [0.5–2.4] cm H_2_O/L, *p* = 0.004). The P_L_ (28.0 [23.5–50.6] to 36.2 [19.6–57.0] cm H_2_O, *p* = 0.6), R_L_ (2.9 [1.8–3.4] to 3.3 [1.8–3.5] cm H_2_O/L/s, *p* > 0.99) and E_L_ (2.5 [1.2–3.2] to 2.8 [1.3–3.8] cm H_2_O/L, *p* > 0.99) remained unchanged with the turnout. Similarly to the preliminary phase, *n* = 3/8 horses had normal lung function after exercise.

#### 3.3.3 Bronchodilation

In this phase, salbutamol was administered 15 min post‐intervention only in the turnout group, to allow comparison between β2‐agonist‐ and exercise‐induced bronchodilation. Compared to the post‐turnout measurements, inhalation of salbutamol improved P_L_ (36.2 [19.6–57.0] to 12.6 [11.1–17.7] cm H_2_O, *p* = 0.006), R_L_ (3.3 [1.8–3.5] to 1.2 [1.0–1.8] cm H_2_O/L/s, *p* = 0.008) and E_L_ (2.8 [1.3–3.8] to 0.8 [0.7–1.3] cm H_2_O/L, *p* = 0.03). After the exercise intervention, P_L_ (16.2 [10.2–35.2] to 17.2 [9.6–28.8] cm H_2_O, *p* = 0.2), R_L_ (1.3 [0.8–2.2] to 1.3 [0.7–2.4] cm H_2_O/L/s, *p* = 0.7) and E_L_ (0.9 [0.5–2.4] to 1.0 [0.5–2.4] cm H_2_O/L, *p* = 0.7) remained unchanged from 15 to 20 min post‐exercise (Figure 2). There were no differences between interventions in lung function parameters at 20 min, indicating that the bronchodilation induced by exercise was similar to that obtained with an inhaled β2‐agonist.

## 4 DISCUSSION

A short, low‐intensity exercise induced clinically relevant bronchodilation in horses experiencing clinical exacerbation of SEA, reducing R_L_ and E_L_ by approximately 50% in both study phases. Furthermore, the degree of bronchodilation obtained after exercise was similar to that achieved with the inhalation of a β2‐agonist, a finding also reported using an anticholinergic bronchodilator (inhaled ipratropium).^14^ This degree of lung function improvement is comparable to that seen in healthy horses, where airway resistance decreases by 45%–70% after exercise, depending on the training protocols.^9^, ^10^, ^17^

Despite its high prevalence and consequences for athletic careers and performance,^18^, ^19^ few studies have assessed the impact of exercise on airway obstruction in horses with asthma. The exercise‐induced bronchodilation observed in the current study is consistent and extends previous findings that intense exercise reduces pulmonary resistance by approximately 60% in horses during an acute exacerbation of SEA (4–24 h of antigenic exposure).^13^ Similarly, treadmill exercise performed until fatigue led to a >60% improvement in resistance lasting >10 min post‐exercise in horses with SEA in exacerbation.^14^ Our results demonstrate that exercise‐induced bronchodilation occurs even during more chronic exacerbations and that mild‐intensity exercise produces a significant bronchodilatory response, which lasts at least 20 min after exercise. While exercise‐induced bronchodilation seems consistent in SEA, the response to exercise in milder forms of asthma is uncertain. High‐intensity training in Thoroughbred racehorses led to bronchodilation in healthy animals, but not in horses with mild–moderate asthma.^10^ Whether these apparent discrepancies are caused by methodological factors or different equine asthma phenotypes requires further investigation.

Several physiological mechanisms may have contributed to the improvement in lung function following exercise in SEA. In healthy humans, deep inspirations reduce bronchial tone,^20^ which is a presumed consequence of the actomyosin cross‐bridging disruption in airway smooth muscle during airway distension.^21^ Although the occurrence of larger respiratory cycles has been described in horses at rest and during exercise,^22^ whether it has a bronchodilatory effect in health and asthma in the equine species is unexplored. Of note, the bronchodilation associated with deep inhalations is impaired or abolished in human asthmatics.^23^, ^24^ Another factor that might have contributed to the exercise‐induced bronchodilation is the stress and inhibition of vagal tone, as binding of catecholamines to the β2‐adrenoreceptors of airway smooth muscle results in bronchial relaxation.^25^ However, this mechanism is likely insufficient to explain the degree of bronchodilation achieved by exercise, as the training was mild. Bronchodilation may also have occurred due to the accumulation of CO_2_ in the alveolar space or blood, which has been shown to relax airways in other species,^26^ although this is controversial.^27^ This mechanism may be particularly relevant in horses that can become markedly hypercapnic during exercise, notwithstanding disease status.^28^ Finally, other mechanisms that could have improved lung function include the recruitment of upper airway muscles and pharyngeal opening, improved mucus clearance,^29^ recruitment of closed airways by increased tidal volume, increase in deep body temperature,^30^ and the release of bronchodilator mediators such as nitric oxide and prostaglandins.^31^ However, these mechanisms have been described in humans, and because the respiratory tract response to exercise differs between equine and human asthma, species‐specific studies are needed to identify which are most relevant in horses.

A key finding of the current study is the good tolerability of mild aerobic exercise, as evidenced by horses performing subjectively willingly, the absence of lactate increase, and a return to baseline respiratory and heart rates within 15 min post‐exercise. This is consistent with other studies, in which low‐intensity exercise was generally well tolerated in horses with asthma, even during disease exacerbation.^13^, ^32^ Although cough was not objectively quantified during exercise, it was noted in some horses in the main study; however, it did not interfere with the lungeing exercise, and these horses exhibited a comparable bronchodilator response. Others described a marked distress in SEA horses during exercise, to the extent that some horses collapsed when asked to continue working.^33^ This was not observed in the current cohort, perhaps because the intensity of exercise was mild and the degree of exacerbation was not severe in most horses. The good tolerability of mild exercise in SEA might be explained by the reported increased unloading of oxygen,^34^ and increase in heart rate^35^ that may partially compensate for the hypoxemia that may worsen with exercise in equine asthma.^13^, ^33^, ^34^ Indeed, a light 20‐min trot exercise in SEA horses worsened hypoxemia in the recovery period, but improved PaCO_2_,^33^ likely reflecting enhanced ventilation without a parallel increase in alveolar perfusion, thereby aggravating the pre‐existing ventilation/perfusion mismatch.^36^ Assessment of arterial blood gases would have provided valuable complementary data in the current study, particularly given that exercise‐induced hypoxemia has not been a consistent finding across studies.^37^, ^38^ This variability may be attributable to the small sample sizes and heterogeneity of exercise protocols employed in previous investigations.

Although this study strongly supports that mild exercise has potent bronchodilator properties, some limitations must be considered. First, only the immediate effect of exercise on lung function was assessed. As human asthmatics can also display delayed bronchoconstriction (4–9 h) post‐exercise,^39^ this should be assessed in future studies, along with the duration of bronchodilation. Our study was performed during the spring months under moderate climatic conditions. It is possible that exercise‐induced bronchodilation would not have occurred at colder temperatures, as reported in healthy horses.^40^, ^41^ As the horses' upper airway is less efficient than humans' at warming ambient air,^42^ assessing the impact of cold‐air exercise in SEA is warranted. Another limitation is the lack of cardiovascular assessment in our horses, although heart rate remained within expected values during exercise. As horses with SEA can have right ventricular dysfunction^43^ and have higher pulmonary artery pressure during exercise compared to controls,^37^ they might be at higher risk of cardiovascular events at work, and this should be further investigated before recommending broadly exercise in this population. The potential impact of the weight of a saddle, rider, and the constricting effect of a girth on respiration were not assessed, so future studies should also determine whether the current results apply to ridden exercise. In humans, wearing a corset modifies lung function in healthy and asthmatic individuals.^24^ We also did not objectively assess the velocity of the work, as horses were allowed to adopt their preferred pace. In future studies, pace could be monitored to determine whether there is an association between the degree of work and the amplitude of the bronchodilation. Nonsteroidal anti‐inflammatory drugs (NSAIDs) do not modify baseline lung function in SEA and healthy horses.^44^, ^45^, ^46^ However, phenylbutazone has been shown to attenuate histamine‐induced bronchoconstriction in healthy horses, possibly by inhibiting thromboxane A_2_ release.^46^ Furthermore, in a small cohort of asthmatic humans, acetylsalicylic acid was found to block exercise‐induced bronchodilation,^47^ possibly by inhibiting prostaglandin release. Nonetheless, a clinically relevant bias caused by phenylbutazone administration appears unlikely in the current study, as the magnitude of exercise‐induced bronchodilation was similar between the main study, in which all horses received phenylbutazone, and the preliminary phase, in which only one horse did. Whether lameness‐associated pain may have influenced lung function in the lame horses remains unknown; however, this is considered unlikely, as NSAIDs were administered and their heart rates during exercise were not elevated, suggesting minimal pain. The small sample size and the advanced age of the horses studied also limit the generalisability of the results. Finally, blinding was not attempted due to the obvious external effects of exercise in horses, representing a limitation that was mitigated using objective, standardised lung function assessments.

Horse owners perceive that equine asthma has an important burden on comfort and activity.^1^ Approximately 50% of survey respondents declared that the inability of a horse to continue its current activity would have an impact on their euthanasia decision‐making.^1^ The current study demonstrates that short aerobic exercise feasible in field conditions produces a clinically significant bronchodilation in SEA horses during exacerbation. This finding highlights the need for further research to determine whether exercise can improve respiratory and overall health in affected horses, and whether a structured training program could reduce airway inflammation and remodelling, as reported in rodent models of asthma.^48^, ^49^

## FUNDING INFORMATION

This project was internally funded by the Equine Asthma Research Laboratory (residual funds from different lectures given by Jean‐Pierre Lavoie).

## CONFLICT OF INTEREST STATEMENT

The authors declare no conflicts of interest.

## AUTHOR CONTRIBUTIONS

**Sophie Mainguy‐Seers:** Conceptualization; investigation; writing – original draft; methodology; validation; writing – review and editing; formal analysis; supervision. **Sarah‐Maude Grondin:** Investigation; writing – review and editing. **Jean‐Pierre Lavoie:** Conceptualization; funding acquisition; writing – review and editing; methodology; supervision.

## DATA INTEGRITY STATEMENT

Sophie Mainguy‐Seers and Jean‐Pierre Lavoie had full access to the data and take responsibility for the integrity and accuracy of the data analysis.

## ETHICAL ANIMAL RESEARCH

All experimental procedures were approved by the Animal Care Committee of the Faculty of Veterinary Medicine of the Université de Montréal on 15 March 2024 (Protocol No. 24‐Rech‐2272).

## INFORMED CONSENT

Not applicable.

## Data Availability

The data that support the findings of this study are openly available in the UdeM Dataverse repository at https://doi.org/10.5683/SP3/LT1CAE.
